# Self-Reported Health as Predictor of Allostatic Load and All-Cause Mortality: Findings From the Lolland-Falster Health Study

**DOI:** 10.3389/ijph.2024.1606585

**Published:** 2024-02-01

**Authors:** Neda Esmailzadeh Bruun-Rasmussen, George Napolitano, Stig Egil Bojesen, Christina Ellervik, Knud Rasmussen, Elsebeth Lynge

**Affiliations:** ^1^ Center for Epidemiological Research, Nykøbing Falster Hospital, Nykøbing Falster, Denmark; ^2^ Department of Public Health, University of Copenhagen, Copenhagen, Denmark; ^3^ Department of Clinical Biochemistry, Herlev and Gentofte Hospital, Copenhagen, Denmark; ^4^ Faculty of Health and Medical Sciences, University of Copenhagen, Copenhagen, Denmark; ^5^ Data and Development Support, Region Zealand, Sorø, Denmark; ^6^ Department of Laboratory Medicine, Boston Children’s Hospital and Harvard Medical School, Boston, MA, United States

**Keywords:** allostatic load, self-reported health, mortality, biomarkers, all-cause mortality

## Abstract

**Objectives:** The aim was to determine the association between self-reported health (SRH), allostatic load (AL) and mortality.

**Methods:** Data derived from the Lolland-Falster Health Study undertaken in Denmark from 2016–2020 (*n* = 14,104). Median follow-up time for death was 4.6 years where 456 participants died. SRH was assessed with a single question and AL by an index of ten biomarkers. Multinomial regression analysis were used to examine the association between SRH and AL, and Cox regression to explore the association between SRH, AL and mortality.

**Results:** The risk of high AL increased by decreasing level of SRH. The ratio of relative risk (RRR) of having medium vs. low AL was 1.58 (1.11–2.23) in women reporting poor/very poor SRH as compared with very good SRH. For men it was 1.84 (1.20–2.81). For high vs. low AL, the RRR was 2.43 (1.66–3.56) in women and 2.96 (1.87–4.70) in men. The hazard ratio (HR) for all-cause mortality increased by decreasing SRH. For poor/very poor vs. very good SRH, the HR was 6.31 (2.84–13.99) in women and 3.92 (2.12–7.25) in men.

**Conclusion:** Single-item SRH was able to predict risk of high AL and all-cause mortality.

## Introduction

Self-reported health (SRH) is one of the most frequently used measures of health perception in survey research studies [1]. It is based on a single question (e.g., “Overall, how would you rate your health?”) with a four- or five-point answering scale ranging from “very poor” to “very good.” SRH has consistently been associated with both lifestyle factors, e.g., physical activity, overweight/obesity, smoking, diet, alcohol consumption [1–3], and with the occurrence of diseases, e.g., cardiovascular disease (CVD), lung disease, arthritis, metabolic disease [4–7]. Furthermore, numerous studies have found SRH to be a strong predictor of all-cause mortality [8–10]; and in search for a causal pathway, studies have explored underlying factors that may influence both objectively measured health and a person’s subjective health rating including gender, age, socioeconomic status, and ethnicity [11–13]. Kaplan et al. were the first to examine how the correlation between SRH and mortality was attenuated when controlling for subclinical conditions and disease. They concluded that SRH levels mainly reflect underlying disease burden, and accordingly predict mortality [14]. Later studies have confirmed these findings and additionally reported that SRH influences health-related behaviour that affects outcome, reflects coping resources, and encompass a health awareness not captured by the specific health indicators but relevant to the overall health rating [10, 15–17].

Several studies have also found a strong correlation between SRH and objective health measures [18–22]. Kananen et al. investigated the association of SRH with 150 biomarkers from blood and urine and found 57 of these biomarkers to be associated with SRH, independently of disease and physical functioning in half of the cases [18].

The impact of psychosocial health on SRH including adverse exposures throughout the life course on long-term adult health outcomes have been reported and conceptualized by Erikson and in social determinants of health models [23]. In order to comprehend the potential of SRH as a measure of health in research and clinical practice, it is important to further explore how SRH reflects the condition of the human body [24–26].

Links have been revealed between SRH and allostatic load (AL), defined by cumulative strain of the body due to chronic fluctuation of neural and neuroendocrine responses [26]. AL derives from the concept of allostasis, which is a dynamic regulatory process where homeostasis is maintained through adaptation in the presence of physical and behavioral stressors. The concept was introduced by McEwan and Stellar in 1993 [26]. AL cannot be directly measured but it can be estimated using an AL index, a composite of biomarkers deriving most commonly from the endocrine, metabolic, cardiovascular, and inflammatory systems [27]. AL has been associated with various health outcomes also related to SRH, including CVD, metabolic disease, and all-cause mortality [27, 28]. In our previous study we found a strong correlation between high AL and all-cause mortality; this association was stronger for the AL index than the association between any individual biomarker and all-cause mortality indicating that incorporating risks at the entire range of biomarkers may yield the best prediction of health in general [29].

The association between AL and SRH has been explored in a few studies with methodological limitations including small sample sizes and use of data from only elderly people [20–22]. Therefore, to explore whether SRH has a biologic foundation, we investigated the relation between SRH and AL, and all-cause mortality in the following years in the general adult population from the Lolland-Falster region of Denmark.

## Methods

### Study Population

We used data from the Lolland-Falster Health Study (LOFUS), a household-based population study, initiated to gain knowledge on determinants of health of the inhabitants of Lolland-Falster [30]. Persons aged 18 years and above were randomly sampled from the Danish Civil Registration System and invited to participate together with their household members of all ages. Data collection took place in 2016–2020, and 36% of invited persons participated [31]. A detailed study protocol, and information on socio-economic determinants of participation have been published previously [30, 31]. The present study was restricted to men and non-pregnant women of age 18 and above.

### Health Examinations

Body mass index (BMI) was determined by calculating the ratio of weight to height squared (kg/m^2^) and categorized into four groups: underweight (BMI < 18.5), normal weight (BMI: 18.5–25), overweight (BMI: 25–30) and obesity (BMI > 30) [32]. Waist-to-hip ratio (WHR) was calculated by waist-circumference divided by hip-circumference. WHR was considered high for women with WHR ≥ 0.85 and for men with a WHR ≥ 0.90. Data on blood pressure (BP) was based on three consecutive digital measurements on the upper left arm (apparatus type Welch Allyn Connex pro BPO 3400). Systolic and diastolic blood pressure were calculated as the mean of the second and third measurements. Only one measurement was used if the other was missing.

### Blood Sample Biomarkers

Blood samples were collected in the non-fasting state between 8:40 a.m. and 6:30 p.m. and analyzed at the same day at Department of Clinical Biochemistry at Nykøbing Falster Hospital, accredited by the standard ISO 15189. All instruments were calibrated according to the manufacturer’s instructions [30].

### Allostatic Load

AL was defined consistently with previous studies and in accordance with the initial definition of AL by Seeman et al., 1997 [27, 33]; ten biomarkers were available for the present study representing the; inflammatory system (C-reactive protein (CRP), albumin); metabolic system (high-density lipoprotein cholesterol (HDL-c), low-density lipoprotein cholesterol (LDL-c), triglycerides, glycated haemoglobin (HbA1C), WHR); and the cardiovascular system: (systolic blood pressure (SBP), diastolic blood pressure (DBP), pulse rate (PR).

In the calculation of AL, each biomarker was dichotomized into high risk versus low risk based on quartiles according to age (≤/> 60 years) and sex in line with previous study [27, 28]. The association between each biomarker and all-cause mortality defined whether to use the upper or lower quartile; for most biomarkers, the mortality increased by increasing level of the biomarker, except for HDL-c and albumin known to be inversely related to health outcome. For LDL-c, SBP and DBP a U-shaped association was found. Therefore both the upper and lower quartiles of these biomarkers were denoted high risk values. High-risk biomarkers were assigned a value of 1 and low-risk biomarkers a value of 0, and the AL-index was calculated by summing these scores with equal weights [27, 28]. See Supplementary Table S1. We categorized the total population into three groups based on tertiles of the AL index. “Low AL” included individuals with a summary AL score of 0–2, “medium AL” as 3–4, and “high AL” as 5–10 in line with previous studies [33–35].

### Self-Reported Data

Shortly before the health examination and biological sampling, participants filled in the web-based question: “How would you describe your health in general?”, with the response categories of “very good,” “good,” “fair,” “poor” and “very poor.” The two latter categories were merged in the present analysis and denoted “poor/very poor” as only a few participants reported very poor SRH. Furthermore, data on the presence of chronic conditions (CVD, diabetes and cancer), smoking (never, former, current), and education level (low, medium, high) was included.

### All-Cause Mortality

LOFUS participants were followed-up for death by the Civil Registration System until the 10th of February 2023.

### Data Management and Statistical Analyses

**FIGURE 1:**
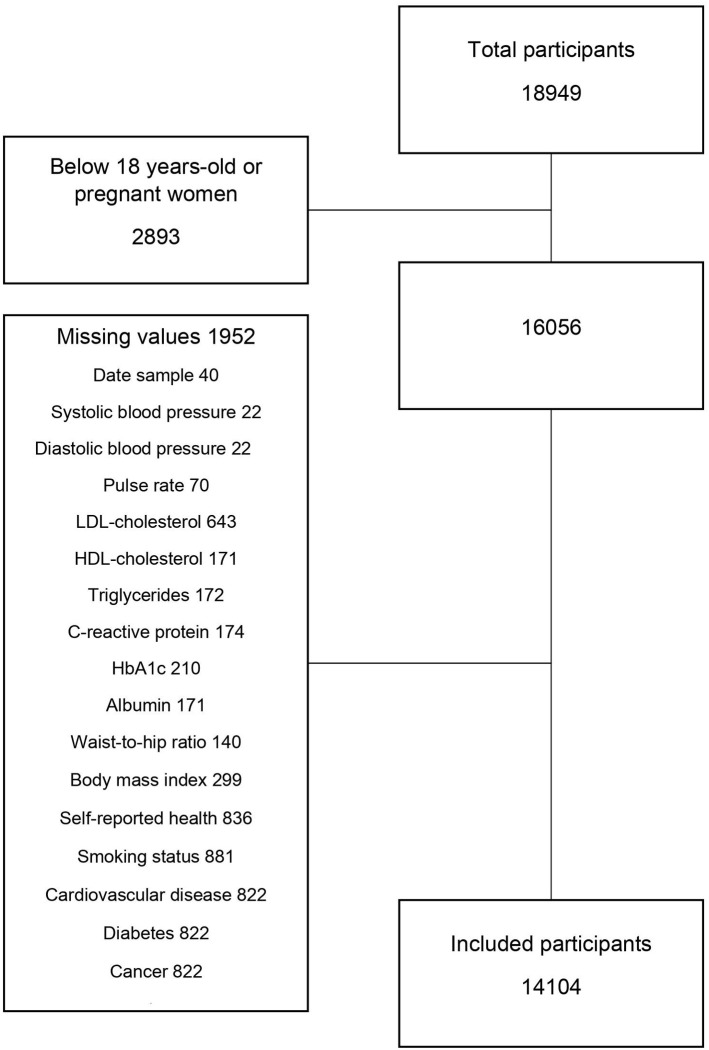
Construction of dataset (Lolland-Falster Health Study, Denmark, 2016–2023).

Numeric values of biomarkers below the limit of detection [L × 10^(−n)^] were replaced with random numbers sampled with replacement from the set {k × 10^(−n)^, k = 1, …, L}, where n is the variable-specific number of decimals reported in the data. Only participants with complete data were included in the analyses (excluded 1,952 out of 16,056, 12.2%, see Figure 1). However, women with missing pregnancy status were included, since they were mostly 50+ years old (84%); moreover, missing education level was classified as “low” as according to the standard language in Denmark used in the questionnaires from LOFUS, no information on education is equal to the fact that the person have the compulsory education only.

To control for possible bias due to missing data, a sensitivity analysis was performed by running a multiple imputation model by chained equations.

Included participant were followed-up from date of participation in the LOFUS study until date of death or end of follow-up on 10 February 2023, whichever came first. Hazard ratios (HR) and corresponding 95% confidence intervals (CI) were obtained from Cox proportional hazard regression models, with time-on-study as time scale. Ratio of relative risks (RRR) and 95% CIs were obtained from multinomial logistic regression models. All models were adjusted for age at baseline (continuous) and sex, and included interaction terms between sex and main predictor, and sex and age. To limit the possible impact of confounding from other factors associated with both SRH and AL, we further adjusted for BMI (spline with 3 degrees of freedom), self-reported education level, smoking status, and presence of cardiovascular diseases, diabetes and cancer as these were the best available intermediate variables in LOFUS.

**FIGURE 2:**
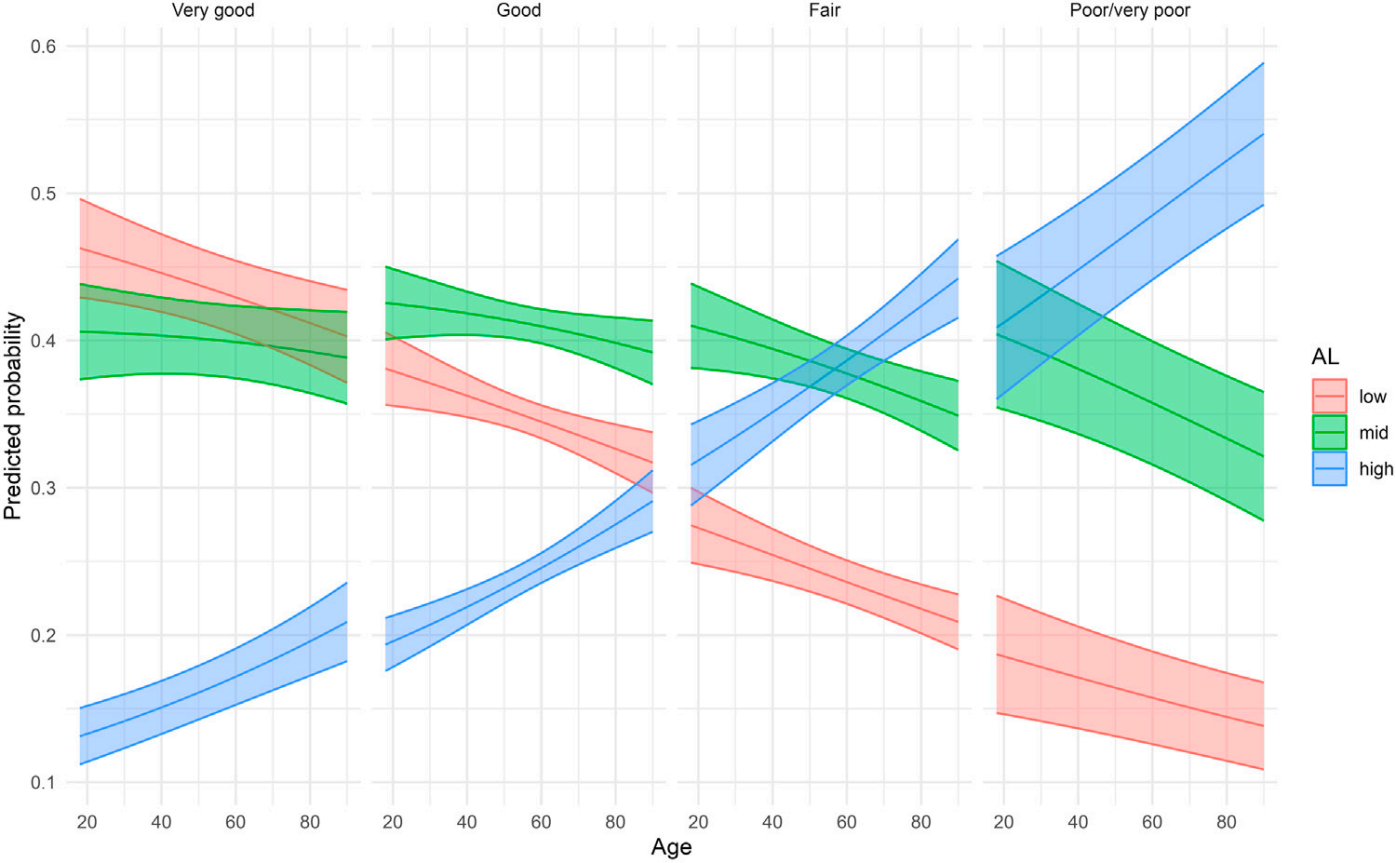
Predicted probabilities of low, medium, and high allostatic load by self-reported health averaged over sex (Lolland-Falster Health Study, Denmark, 2016–2023).

Predicted probabilities of low/medium/high allostatic load, shown in Figure 2, were obtained from the age and sex adjusted multinomial models describe above.

Ratio of predicted probabilities presented in Supplementary Table S2 were computed as estimated marginal means from a multinomial logistic model with SRH as predictor, adjusted for age (categorical) and including interaction terms between sex and SRH, and sex and age.

All analyses and plots were done with R version 4.2.2 [36], with packages tidyverse [37], splines [36], nnet [38], survival [39], emmeans [40].

## Results

A total of 14,104 participants were included (54.1% women and 45.9% men) (Table 1). Poor/very poor SRH was reported by 4.2% (4.5% in women and 3.8% in men) (Supplementary Table S3). One-fourth of the participants had a higher education, and one-fifth were current smokers. Presence of diabetes at the time of LOFUS participation was reported by 5% (3.7% women and 6.8% men), cancer by 4% (3.4% women and 4.2% men), and CVD by 28% (25.6% women and 31.0% men). For BMI, 63% of the participants were overweight or obese (56.6% women and 70.4% men). High AL was observed in 28.1% (29.0% women and 27.1% men), mid AL in 39.8% (39.5% women and 40.2% men) and low AL in 32.1% (31.5% women and 32.7% men). During a median follow-up time of 4.6 years, 456 participants died (189 women and 267 men); 126 were excluded due to missing data (Supplementary Table S4).

**TABLE 1:** Baseline characteristics of the study population according to self-reported health and other variables (Lolland-Falster Health Study, Denmark, 2016–2023).

Variable	Total *n* (%)	Very good *n* (%)	Good *n* (%)	Fair *n* (%)	Poor/very poor *n* (%)	Death *n* (%)
Total	14,104 (100)	1,749 (100)	8,077 (100)	3,688 (100)	590 (100)	456 (100)
Men	6,474 (45.9)	849 (48.5)	3,755 (46.5)	1,627 (44.1)	243 (41.2)	267
Women	7,630 (54.1)	900 (51.5)	4,322 (53.5)	2,061 (55.9)	347 (58.8)	189
Age						
18–29	1,023 (7.3)	220 (21.5)	610 (59.6)	169 (16.5)	24 (2.3)	10 (2.2)^a^
30–39	1,163 (8.2)	129 (11.1)	714 (61.4)	281 (24.2)	39 (3.4)	^a^
40–49	2,098 (14.9)	278 (13.3)	1,234 (58.8)	495 (23.6)	91 (4.3)	^a^
50–59	3,136 (22.2)	342 (10.9)	1,728 (55.1)	884 (28.2)	182 (5.8)	43 (9.4)
60–69	3,593 (25.5)	411 (11.4)	2,048 (57.0)	985 (27.4)	149 (4.1)	108 (23.7)
70–79	2,513 (17.8)	278 (11.1)	1,428 (56.8)	720 (28.7)	87 (3.5)	163 (35.7)
+80	578 (4.1)	91 (15.7)	315 (54.5)	154 (26.6)	18 (3.1)	132 (28.9)
Allostatic load						
Low (0–2)	4,522 (32.1)	759 (16.8)	2,801 (61.9)	867 (19.2)	95 (2.1)	94 (20.6)
Medium (3–4)	5,618 (39.8)	698 (12.4)	3,313 (59.0)	1,397 (24.9)	210 (3.7)	169 (37.1)
High (5–10)	3,964 (28.1)	292 (7.4)	1,963 (49.5)	1,424 (35.9)	285 (7.2)	193 (42.3)
Education						
Low	3,403 (24.1)	374 (11.0)	1,724 (50.7)	1,096 (32.2)	209 (6.1)	157 (34.4)
Medium	7,024 (49.8)	812 (11.6)	4,128 (58.8)	1,805 (25.7)	279 (4.0)	222 (48.7)
High	3,677 (26.1)	563 (15.3)	2,225 (60.5)	787 (21.4)	102 (2.8)	77 (16.9)
Smoking status						
Never	6,503 (46.1)	1,020 (15.7)	3,921 (60.3)	1,391 (21.4)	171 (2.6)	121 (26.5)
Former	4,896 (34.7)	534 (10.9)	2,741 (56.0)	1,399 (28.6)	222 (4.5)	219 (48.0)
Current	2,705 (19.2)	195 (7.2)	1,415 (52.3)	898 (33.2)	197 (7.3)	116 (25.4)
Body mass index						
Under Weight (<18.5)	182 (1.2)	29 (15.9)	86 (47.3)	54 (29.7)	13 (7.1)	10 (2.2)
Normal weight (18.5–24.9)	5,041 (35.7)	891 (17.7)	3,046 (60.4)	973 (19.3)	131 (2.6)	158 (34.6)
Over Weight (25.0–29.9)	5,404 (38.3)	636 (11.8)	3,235 (59.9)	1,337 (24.7)	196 (3.6)	177 (38.8)
Obese (>30.0)	3,477 (24.7)	193 (5.6)	1,710 (49.2)	1,324 (38.1)	250 (7.2)	111 (24.3)
Cardiovascular disease						
Yes	3,961 (28.1)	1,491 (14.7)	6,058 (59.7)	2,264 (22.3)	330 (3.3)	234 (51.3)
No	10,143 (71.9)	258 (6.5)	2,019 (51.0)	1,424 (36.0)	260 (6.6)	222 (48.7)
Diabetes						
Yes	723 (5.1)	30 (4.1)	292 (40.4)	334 (46.2)	67 (9.3)	53 (11.6)
No	13,381 (94.9)	1,719 (12.8)	7,785 (58.2)	3,354 (25.1)	523 (3.9)	403 (88.4)
Cancer						
Yes	532 (3.8)	28 (5.3)	238 (44.7)	224 (42.1)	42 (7.9)	74 (16.2)
No	13,572 (96.2)	1,721 (12.7)	7,839 (57.8)	3,464 (25.5)	548 (4.0)	382 (83.8)

Note: Persons with missing value on at least one variable are excluded, see Figure 1 and Supplementary Table S4.

^a^ For the age group 18–49 the number of deaths were combined as the groups were very small.

The association between SRH and all-cause mortality was investigated in a multivariate Cox proportional hazard regression, Table 2. Compared with the mortality of persons who had reported very good health, the HRs for death was for women statistically significantly elevated for all other levels of SRH; good SRH, HR 2.23 (95% CI 1.12–4.42); fair SRH, HR 2.42 (95% CI 1.20–4.90; and poor/very poor SRH, HR 6.31 (95% CI 2.84–13.9). For men a statistically significant increase in mortality was seen for fair SRH, HR 2.20 (95% CI 1.32–3.66) and for poor/very poor SRH, HR 3.92 (95% CI 2.12–7.25).

**TABLE 2:** Multivariate Cox proportional hazard regression of all-cause mortality for Lolland-Falster Health Study participants (Lolland-Falster Health Study, Denmark, 2016–2023).

Variable	Value	Women		Men	
		HR 1 (95% CI)	HR 2 (95% CI)	HR 1 (95% CI)	HR 2 (95% CI)
Self-reported health	Very good	1	1	1	1
	Good	2.40 (1.21–4.75)	2.23 (1.12–4.42)	1.42 (0.86–2.33)	1.25 (0.76–2.06)
	Fair	3.10 (1.54–6.22)	2.42 (1.20–4.90)	2.91 (1.77–4.80)	2.20 (1.32–3.66)
	Poor/very poor	7.89 (3.59–17.35)	6.31 (2.84–13.9)	6.62 (3.66–11.9)	3.92 (2.12–7.25)
Allostatic load	Low (0–2)	1	1	1	1
	Mid (3–4)	1.36 (0.91–2.02)	1.30 (0.87–1.93)	1.32 (0.95–1.83)	1.25 (0.90–1.74)
	High (5–10)	2.33 (1.59–3.42)	2.40 (1.61–3.57)	2.04 (1.48–2.81)	1.78 (1.27–2.49)

HR 1: Adjusted for age at LOFUS-visit.

HR 2: Further adjusted for education, body mass index, smoking status, cardiovascular disease, diabetes, and cancer.

Compared with the mortality of persons with low AL, women with high AL had increased mortality, HR 2.40 (95% CI 1.61–3.57) and so had men, HR 1.78 (95% CI 1.27–2.40) (Supplementary Figure S1). Mutually adjusted models for SRH, AL and mortality are presented in Supplementary Table S5.

Multivariate Cox proportional hazard regression of all-cause mortality by SRH and individual biomarkers, mutually adjusted are presented in Supplementary Table S6. The impact of the biomarkers were weak for most biomarkers when adjusting for SRH; except for LDL and WHR in women HR 1.70 (95% CI 1.25–2.32) and HR 1.59 (1.15–2.20), respectively, and for albumin in men HR 1.83 (95% CI 1.44–2.33).

Multinomial regression models for SRH as predictor of AL for women and men are presented in Table 3. In women the risk of having medium as compared with low AL increased with decreasing level of reported SRH. In the multivariate adjusted model, RRR of medium vs. low AL was 1.23 (95% CI 1.04–1.44) in women reporting good as compared with very good SRH; RRR 1.52 (95% CI 1.26–1.84) in fair compared with very good SRH; and RRR 1.58 (95% CI 1.11–2.23) in poor/very poor compared with very good SRH. The pattern was less clear for men, and only the RRR for medium vs. low AL in men reporting poor/very poor as compared very good SRH reached statistical significance, RRR 1.84 (95% CI 1.20–2.81). For high vs. low AL, the RRR increased more sharply across levels of SRH. In the multivariate adjusted model for women, the RRR for good vs. very good SRH was 1.34 (95% CI 1.08–1.67); for fair vs. very good SRH it was RRR 2.03 (95% CI 1.60–2.58); and for poor/very poor vs. very good SRH it was RRR 2.43 (95% CI 1.66–3.56). In this model for men, only the two latter comparisons reached statistical significance, for fair vs. very good SRH, RRR 1.58 (95% CI 1.23–2.03); and for poor/very poor vs. very good, RRR 2.96 (95% CI 1.87–4.70). For each level of SRH, the probability of having high AL increased by increasing age, Figure 2.

**TABLE 3:** Difference by level of self-reported health in risk of increased level of allostatic load, ratio of relative risks (RRR) (Lolland-Falster Health Study, Denmark, 2016–2023).

	Values	Women		Men	
		RRR 1 (95% CI)	RRR 2 (95% CI)	RRR1 (95% CI)	RRR 2 (95% CI)
AL		Medium vs. low	Medium vs. low	Medium vs. low	Medium vs. low
SRH	Very good	1	1	1	1
	Good	1.37 (1.17–1.61)	1.23 (1.04–1.44)	1.19 (1.01–1.40)	1.05 (0.88–1.24)
	Fair	1.98 (1.65–2.38)	1.52 (1.26–1.84)	1.50 (1.23–1.82)	1.11 (0.91–1.36)
	Poor/very poor	2.27 (1.16–3.20)	1.58 (1.11–2.23)	2.65 (1.74–4.01)	1.84 (1.20–2.81)
AL		High vs. low	High vs. low	High vs. low	High vs. low
SRH	Very good	1	1	1	1
	Good	2.01 (1.64–2.47)	1.34 (1.08–1.67)	1.57 (1.27–1.95)	1.08 (0.87–1.36)
	Fair	4.67 (3.75–5.83)	2.03 (1.60–2.58)	3.59 (2.85–4.53)	1.58 (1.23–2.03)
	Poor/very poor	7.66 (5.40–10.86)	2.43 (1.66–3.56)	7.82 (5.11–11.97)	2.96 (1.87–4.70)

AL, allostatic load. SRH, self-reported health.

RRR 1: Adjusted for age at baseline.

RRR 2: Further adjusted for education, body mass index, smoking status, cardiovascular disease, diabetes, and cancer.

A notable difference was found between the age-adjusted and fully-adjusted model for RRR of high vs. low AL for poor/very poor SRH in women and men. We therefore further explored the intermediate variables in a multinomial model and found that when adjusting for BMI the association between AL and SRH attenuated more than when adjusting for the other variables (Supplementary Table S7). Finally, we made sensitivity analysis by investigating the association between AL and mortality and SRH and AL by using clinical cut-off values to define AL. The AL categories were slightly changed to low AL: 0–2, medium AL: 3, and high AL: 4–9, as no participants had AL10 (Supplementary Tables S8, S9). We found mostly a weaker association between AL and SRH, and also AL and mortality when comparing with AL based on the distribution of our population.

The distribution of biomarkers according to self-reported health are presented in (Supplementary Table S10).

## Discussion

In the general population study from Lolland-Falster in Denmark, SRH was strongly associated with AL. Despite SRH being an instant and subjective assessment of overall health; the relative risk of harbouring high vs. low AL at the same time was more than doubled among both women and men reporting poor/very poor health as compared with those reporting very good health, even when controlling for several known risk factors. Additionally, after follow-up of the study population for a median of 4.6 years, SRH was found to be strongly associated with all-cause mortality even after control for known risk factors. Women reporting poor/very poor SRH had 6-fold the death rate of those reporting very good SRH and for men, an almost 4-fold ratio was seen.

Among the 14,104 LOFUS-participants included in the study, 4.2% reported poor/very poor SRH. This was higher than the 1.4% reported in the Copenhagen Aging and Midlife Biobank study, comprising of participants from the Metropolit 1953 Danish Male Birth Cohort, the Copenhagen Perinatal Cohort, and the Danish Longitudinal Study on Work, Unemployment and Health cohort [41]. In Europe, reporting of poor SRH in the adult population has decreased during the last decades, and poor SRH has been most prevalent in lower socioeconomic classes [2, 42, 43]. The higher rate of poor SRH in LOFUS than in other Danish cohorts may be linked to the fact that Lolland-Falster is a socio-economically disadvantaged area of Denmark, with health problems reported more frequently, and with shorter life expectancy than the rest of Denmark [44].

We found that the association between SRH and AL was stronger when the AL was calculated using our own cut-off values, compared to AL based on clinical defined cut-off values. This might be a consequence of the fact that in our population clinical cut-offs tend to produce a generally lower AL, smoothing down the differences between the AL categories. The number of studies exploring the relationship between SRH and AL is sparse. Hasson et al. found in a cross-sectional study, that poor SRH, along with older age, low education, and work in healthcare as compared with work in information technology was associated with higher AL in Swedish women [20]. These findings were supported by the Norwegian HUNT study further indicating level of SRH in adolescence to be a predictor of AL in young adulthood [21]. Among elderly persons in Taiwan, Hu et al. found SRH to be related to AL [22], contributing valuable evidence of construct validity of AL in a non-Western population as most studies on AL are of Western orientation. A more recent study from the United States used cross-sectional data from the National Health and Nutrition Examination Survey (NHANES) and also found higher levels of AL to be associated with higher odds of reporting poor/fair SRH [45]. This association was found to differ by race/ethnicity.

When evaluating the intermediate variables separately, we found that the association between SRH and AL was primarily attenuated by BMI, a well-established risk factor for numerous diseases and all-cause mortality [32].

Our findings on the association between SRH and mortality is also consistent with previous studies [8–10]; in a systematic review, Idler et al. found a strong correlation between SRH and all-cause mortality [9]. Additionally, in a subsequent meta-analysis DeSalvo et al. found that people with poor SRH had a two-fold mortality risk when comparing with those reporting very good SRH [46]. Studies from Europe have reported a similar association between SRH and all-cause mortality in both adults and in older populations [47–50]. In a comprehensive analysis conducted by Parker et al., encompassing a systematic review and meta-analysis of 17 studies, it was concluded that despite significant variations in the ages of the participants, AL indices, and follow-up time, still a notable association existed between high AL and elevated risk of mortality [28]. The pooled estimates indicated that individuals with high AL had a 22% higher risk of all-cause mortality and a 31% higher risk of cardiovascular mortality compared to those with low AL. In the majority of studies, the most substantial correlation with mortality was observed when incorporating biomarkers that captured all four organ systems. In our data, we found an almost two-fold mortality in persons with high AL as compared with those with low AL when using our own AL construct. It should however be taken into consideration that the construct of our AL index was slightly different than in other studies due to the fact that both the upper and lower quartiles for some biomarkers defined high risk values [27]. This may also explain the stronger association found when using cut off-values based on the population to define AL when comparing with AL based on cut-off values.

SRH is a subjective summary of the understanding and interpretation of “health,” and it might be influenced by several factors; 1) direct information of one’s disease, severity, and prognosis; 2) lifestyle and behavioral risk factors including unhealthy diet and smoking; and 3) perceptions and sensations about the body and mind, such as pain and tiredness [19]. It is known that the level of expectations regarding “good” health decreases with increasing age, where more health problems are tolerated at a given level of stated SRH [48].

The present study contributed by studying SRH, AL and mortality in a large population with a wide age span. There are several ways in which AL can have an impact on SRH. First, biomarkers may act as surrogate measures for medical conditions that were not directly measured in this study [47]. Second, biomarkers may reflect an unhealthy lifestyle known to effect SRH [7]. Third, the state of physiologic system may be accessible to individuals through interoception, a system of feelings that represents a sense of the physiologic condition of the body [48–50]. Most studies on the interoceptive signalling of humoral processes and changes in biomarker levels concern inflammation, in which higher circulating levels of inflammatory biomarkers cause fatigue, poor appetite, low mood and general malaise. However, the empirical evidence on interoceptive signalling of humoral processes is still lacking and almost non-existent for biomarkers of other organ systems [48].

Our results contributed to the ongoing debate on the role of SRH in predicting mortality. According to our findings SRH has a biologic basis and may reflect strain on the body in terms of AL. Future research are needed to support our findings and to investigate both the physiological processes that underlie sensations and the reason that governs ones perception about health. Further studies should focus on the pathways that mediate information from the human organism to the consciousness incorporating information into rating ones health.

### Strengths and Limitations

The strengths of the study included the prospective design in a large population-based sample, the follow-up time, and the number of deaths obtained by linkage with the Danish Civil Registration System. It was a strength that our study population covered a wide age-span and that we were able to control for confounders known to be associated with both SRH and AL. It was additionally a strength that we used sex-specific cut points for individual biomarkers in the construct of AL. However, several limitations should be considered. Firstly, our study was restricted to the biomarkers available and did therefore not include biomarkers from the neuroendocrine system although it is reported that the neuroendocrine system plays a key role in allostasis and subsequent AL. This is due to the fact that a series of physiological changes takes place before initial stress responses occur (including rapid increases in blood sugar levels and blood pressure that supply the body with additional energy). However, biomarkers from the neuroendocrine system are difficult to measure in population studies where participants are examined only once as it is recommended that biomarkers from the neuroendocrine system are measured repeatedly over 1–2 days. Differences across studies in the definition of AL including the choice of biomarkers and their weighing could influence the comparison of results. The operationalization of AL into a single index that represents dysregulation across systems is still of discussion. The most common approach is the count-based method, where a summary score is calculated by summing the number of biomarkers falling within the high-risk category, mostly defined by the percentile (either the upper or lower 25th percentile of the sample’s distribution) or by the clinical cut-off values. Another approach is calculating AL scores individually across different organ systems and then sum the systems-level scores to create an overall AL score. In most cases only increasing or decreasing associations between biomarker value and mortality are taken into account, and this will affect the calculated AL score both when it is analysed as a categorical or a continuous variable. We tried to overcome some of the limitations by considering also u-shaped associations between biomarker values and mortality. Nevertheless, the complexity of identifying an index that adequately reflects the underlying profile of dysregulation remains a challenge. Secondly, we excluded 12% of participants with missing data. In total, 55,144 persons from the general population were invited to participate in LOFUS. Up until the end of February 2023, 3,514 of these persons died. The participation rate in LOFUS was 36%, and the non-participants had an age-standardized mortality rate almost three times that of participants; rate ratio 2.94 (95% CI 2.69–3.22), clearly showing that LOFUS participants constituted a health selected subgroup [31]. Within the LOFUS participants included in the present study, 3.2% (=456/14,104) of the participants with a full data set died, while this was the case for 6.5% (=126/1,952) of the participants with some missing data. Two steps of health selection therefore affected the participants with a full data set included in the analysis. Fortunately, participants with some missing data constituted only a minor proportion of the participants, and a sensitivity analysis based on imputed values for missing data gave results in line with those for participants with full data (Supplementary Tables S11, S12).

Finally, measures of depression and cognition were not included in the analyses.

### Conclusion

Based on data from more than 14,000 adults, we found a strong correlation between SRH, AL and mortality suggesting that SRH has a biological basis surpassing personal differences in perceptions of health.
