# Supplementary material for: Self-Reported Health as Predictor of Allostatic Load and All-Cause Mortality: Findings From the Lolland-Falster Health Study
Source: Int J Public Health. 2024 Feb 1;69:1606585. doi: 10.3389/ijph.2024.1606585 (PMC10866731; doi:10.3389/ijph.2024.1606585)
Supplement: Supplementary file 1 [file Table5.pdf]

Supplementary Table 5. Multivariate Cox proportional hazard regression of all-cause mortality for participants, mutually adjusted

		**HR**<br>**Model 1C**	**HR**<br>**Model 1C + adj**	**HR**<br>**Model 1C**	**HR**<br>**Model 1C + adj**
		**Women**		**Men**	
**Self-reported health**	**Very good**	1	1	1	1
	**Good**	2.25 (1.13 – 4.46)	2.14 (1.08 – 4.24)	1.40 (0.85 – 2.30)	1.26 (0.77 – 2.08)
	**Fair**	2.69 (1.34 – 5.43)	2.22 (1.10 – 4.49)	2.76 (1.68 – 4.56)	2.23 (1.34 – 3.71)
	**Poor/ very poor**	6.53 (2.95 – 14.45)	5.47 (2.45 – 12.20)	5.72 (3.14 – 10.42)	3.74 (2.02 – 6.92)
**Alloastatic load**	Low (0–2)	1	1	1	1
	Mid (3–4)	1.28 (0.86 – 1.91)	1.27 (0.85 – 1.90)	1.19 (0.86 – 1.65)	1.21 (0.87 – 1.69)
	High (5–10)	2.03 (1.37 – 2.99)	2.29 (1.53 – 3.42)	1.69 (1.22 – 2.34)	1.66 (1.18 – 2.34)

Model 1C: Mortality ~ (self-reported health + allostatic load + age)* sex.

Model 1C + adjusted: – further adjusted for education, body mass index (spline), smoking status, cardiovascular disease, diabetes, and cancer.
