# Supplementary material for: Self-Reported Health as Predictor of Allostatic Load and All-Cause Mortality: Findings From the Lolland-Falster Health Study
Source: Int J Public Health. 2024 Feb 1;69:1606585. doi: 10.3389/ijph.2024.1606585 (PMC10866731; doi:10.3389/ijph.2024.1606585)
Supplement: Supplementary file 2 [file Table4.pdf]

Supplementary Table 4. Baseline characteristics of excluded participants

	Excluded participants n (%)	
	**Participants**	**Deaths**
**Total**	1952	126 (6.5)
**Sex**		
Women	847 (43.4)	39 (31.0)
Men	1105 (56.6)	87 (69.0)
**Age**		
18-49	718 (36.8)	3 (2.4)
50-79	1103 (56.6)	79 (62.8)
80+	131 (6.7)	44 (34.9)
**Allostatic load**		
Low (0–2)	257 (13.2)	11 (8.7)
Medium (3–4)	413 (21.2)	24 (19)
High (5–10)	379 (19.4)	43 (34.1)
Missing	903 (46.3)	48 (38.1)
**Education**		
Low	1203 (61.6)	77 (61.1)
Medium	535 (27.4)	34 (27)
High	214 (11)	15 (11.9)
**Smoking status**		
Never	425 (21.8)	22 (17.5)
Former	420 (21.5)	48 (38.1)
Current	226 (11.6)	9 (7.1)
Missing	881 (45.1)	47 (37.3)
**Body mass index**		
Underweight (<18.5)	19 (1.0)	4 (3.2)
Normal weight (18.5–24.9)	435 (22.3)	21 (16.7)
Overweight (25.0–29.9)	638 (32.7)	21 (16.7)
Obese (>30.0)	561 (28.7)	25 (19.8)
Missing	299 (15.3)	55 (43.7)
**Cardiovascular disease**		
Yes	470 (24.1)	54 (42.9)

No	660 (33.8)	31 (24.6)
Missing	822 (42.1)	41 (32.5)
**Diabetes**		
Yes	121 (6.2)	15 (11.9)
No	1009 (51.7)	70 (55.6)
Missing	822 (42.1)	41 (32.5)
**Cancer**		
Yes	73 (3.7)	19 (15.1)
No	1057 (54.1)	66 (52.4)
Missing	822 (42.1)	41 (32.5)
**Self-reported health**		
Very good	599 (30.7)*	27 (21.4)*
Good	*	*
Fair	399 (20.4)	36 (28.6)
Poor/very poor	118 (6.0)	16 (12.7)
Missing	836 (42.8)	47 (37.3)

* For the excluded participants, number of individuals reporting “very good” and “good” health were combined as the groups were very small.
