# Supplementary material for: Self-Reported Health as Predictor of Allostatic Load and All-Cause Mortality: Findings From the Lolland-Falster Health Study
Source: Int J Public Health. 2024 Feb 1;69:1606585. doi: 10.3389/ijph.2024.1606585 (PMC10866731; doi:10.3389/ijph.2024.1606585)
Supplement: Supplementary file 3 [file Table11.pdf]

Supplementary Table 11. Multivariate Cox proportional hazard regression of all-cause mortality for participants including imputed data

Imputation method: Multiple imputation by chained equations.

N participants = 16016 (40 removed because of missing baseline date)

		**HR1 (95% CI)**	**HR2 (95% CI)**	**HR1 (95% CI)**	**HR2 (95% CI)**
		**Women**		**Men**	
**AL**	**Low (0–2)**	1	1	1	1
	**Medium (3–4)**	1.20 (0.84 – 1.73)	1.16 (0.81 – 1.67)	1.32 (0.99 – 1.76)	1.28 (0.96 – 1.7)
	**High (5–10)**	2.09 (1.48 – 2.95)	2.14 (1.49 – 3.07)	2.17 (1.64 – 2.86)	1.88 (1.39 – 2.52)
**SRH**	**Very good**	1	1	1	1
	**Good**	2.40 (1.22 – 4.72)	2.26 (1.14 – 4.48)	1.45 (0.91 – 2.31)	1.27 (0.79 – 2.03)
	**Fair**	3.28 (1.66 – 6.46)	2.61 (1.31 – 5.18)	2.95 (1.84 – 4.71)	2.23 (1.38 – 3.61)
	**Poor/Very poor**	6.57 (3.04 – 14.2)	5.54 (2.53 – 12.17)	6.86 (4.03 – 11.68)	4.35 (2.51 – 7.54)

AL = allostatic load, SRH = self-reported health
HR1: adjusted for age at baseline.
HR2: further adjusted for education, body mass index, smoking status, cardiovascular disease, diabetes, and cancer.
