# Supplementary material for: Self-Reported Health as Predictor of Allostatic Load and All-Cause Mortality: Findings From the Lolland-Falster Health Study
Source: Int J Public Health. 2024 Feb 1;69:1606585. doi: 10.3389/ijph.2024.1606585 (PMC10866731; doi:10.3389/ijph.2024.1606585)
Supplement: Supplementary file 4 [file Table1.pdf]

Supplementary Table 1. High-risk cut points and clinical cut points for individual biomarkers

Characteristics	Women N = 7630			Men N =6474		
	<60 years	≥60 years	Clinical cut points	<60 years	≥60 years	Clinical cut points
HDL-c, mg/dl	≤1.3	≤1.4	≤1.0	≤1.0	≤1.1	≤1.0
LDL-c, mg/dl	≤2.2 or ≥3.4	≤2.4 or ≥3.8	≥2.6	≤2.2 or ≥3.4	≤2.1 or ≥3.5	≥2.6
Triglycerides, mg/dl	≥1.8	≥2	≥2.0	≥2.4	≥2.2	≥2.0
Albumin, g/dl	≤38	≤37	<35	≤39	≤37	<35
C-reactive protein, mg/L	≥3.58	≥3.53	≥3.00	≥2.37	≥3.42	≥3.00
HbA1c, mmol/mol	≥37	≥40	≥42	≥37	≥40	≥42
Systolic blood pressure, mmHg	≤112 or ≥130.5	≤125 or ≥152.5	<90 or ≥140	≤120 or ≥138.5	≤128.5 or ≥153.5	<90 or ≥140
Diastolic blood pressure, mmHg	≤ 72 or ≥83	≤74.5 or ≥83.5	<60 or ≥90	≤73.5 or ≥85	≤75 or ≥85.5	<60 or ≥90
Pulse rate, per minute	≥74	≥74	≥90	≥72	≥72	≥90
Waist-to-hip ratio	≥0.88	≥0.91	≥0.85	≥0.99	≥1.03	≥0.90

HDL-c = High-density lipoprotein cholesterol
LDL-c = Low-density lipoprotein cholesterol
