# Supplementary material for: Self-Reported Health as Predictor of Allostatic Load and All-Cause Mortality: Findings From the Lolland-Falster Health Study
Source: Int J Public Health. 2024 Feb 1;69:1606585. doi: 10.3389/ijph.2024.1606585 (PMC10866731; doi:10.3389/ijph.2024.1606585)
Supplement: Supplementary file 5 [file Table2.pdf]

Supplementary table 2. Ratio of predicted probabilities of allostatic load level by self-reported health (averaged over age-groups) from multinomial model

	**Multinomial logistic model* – RR^ (95% CI)**					
	**Women**			**Men**		
	**Allostatic load**			**Allostatic load**		
**Self-reported health**	**Low (0–2)**	**Medium (3–4)**	**High (5–10)**	**Low (0–2)**	**Medium (3–4)**	**High (5–10)**
**Very good**	Ref	Ref	Ref	Ref	Ref	Ref
**Good**	0.76 (0.7 – 0.83)	1.05 (0.96 – 1.15)	1.54 (1.31 – 1.80)	0.84 (0.77 – 0.92)	1.03 (0.95 – 1.13)	1.30 (1.11 – 1.53)
**Fair**	0.50 (0.45 – 0.56)	1.00 (0.91 – 1.11)	2.35 (2.01 – 2.75)	0.61 (0.55 – 0.69)	0.95 (0.85 – 1.05)	2.09 (1.78 – 2.46)
**Poor/ very poor**	0.39 (0.31 – 0.5)	0.90 (0.76 – 1.06)	2.92 (2.43 – 3.51)	0.37 (0.27 – 0.51)	0.99 (0.83 – 1.18)	2.58 (2.11 – 3.16)
