# Supplementary material for: Self-Reported Health as Predictor of Allostatic Load and All-Cause Mortality: Findings From the Lolland-Falster Health Study
Source: Int J Public Health. 2024 Feb 1;69:1606585. doi: 10.3389/ijph.2024.1606585 (PMC10866731; doi:10.3389/ijph.2024.1606585)
Supplement: Supplementary file 6 [file Table9.pdf]

Supplementary Table 9. Difference by level of self-reported health in risk of increased level of allostatic load defined by clinical cut-off values, ratio of relative risks (RRR)

		RRR1 (95% CI)	RRR2 (95% CI)	RRR1 (95% CI)	RRR2 (95% CI)
		**Women**		**Men**	
**Allostatic load**		**Medium vs. low**	**Medium vs. low**	**Medium vs. low**	**Medium vs. low**
**Self-reported health**	**Very good**	1	1	1	1
	**Good**	1.73 (1.40 – 2.14)	1.38 (1.10 – 1.72)	1.54 (1.26 – 1.88)	1.27 (1.03 – 1.55)
	**Fair**	2.40 (1.91 – 3.02)	1.52 (1.19 – 1.93)	1.91 (1.53 – 2.39)	1.28 (1.02 – 1.62)
	**Poor/ very poor**	3.25 (2.31 – 4.56)	1.71 (1.20 – 2.45)	2.30 (1.55 – 3.43)	1.54 (1.01 – 2.35)
**Allostatic load**		**High vs. low**	**High vs. low**	**High vs. low**	**High vs. low**
**Self-reported health**	**Very good**	1	1	1	1
	**Good**	1.69 (1.35 – 2.11)	1.14 (0.89 – 1.45)	1.36 (1.13 – 1.63)	0.97 (0.80 – 1.18)
	**Fair**	3.39 (2.68 – 4.27)	1.54 (1.19 – 1.99)	2.23 (1.82 – 2.73)	1.11 (0.89 – 1.39)
	**Poor/ very poor**	5.26 (3.81 – 7.28)	1.81 (1.26 – 2.60)	3.53 (2.49 – 4.99)	1.68 (1.14 – 2.49)

RRR 1: Adjusted for age at baseline.
RRR 2: Further adjusted for education, body mass index, smoking status, cardiovascular disease, diabetes, and cancer.
