# Supplementary material for: Self-Reported Health as Predictor of Allostatic Load and All-Cause Mortality: Findings From the Lolland-Falster Health Study
Source: Int J Public Health. 2024 Feb 1;69:1606585. doi: 10.3389/ijph.2024.1606585 (PMC10866731; doi:10.3389/ijph.2024.1606585)
Supplement: Supplementary file 7 [file Table6.pdf]

Supplementary Table 6. Multivariate Cox proportional hazard regression of all-cause mortality for Lolland-Falster Health Study participants by self-reported health and individual biomarkers, mutually adjusted

Variables	Self-reported health	HR (95% CI)	HR (95% CI)
		Women	Men
	Very good	1	1
	Good	2.21 (1.11 – 4.38)	1.25 (0.76 – 2.06)
	Fair	2.39 (1.18 – 4.84)	2.20 (1.32 – 3.66)
	Poor/ very poor	6.13 (2.76 – 13.60)	3.92 (2.12 – 7.25)
Systolic blood pressure	High vs. low risk	1.34 (1.00 – 1.80)	0.98 (0.77 – 1.24)
	Very good	1	1
	Good	2.23 (1.12 – 4.42)	1.25 (0.76 – 2.07)
	Fair	2.41 (1.19 – 4.87)	2.20 (1.32 – 3.67)
	Poor/ very poor	6.36 (2.87 – 14.11)	3.93 (2.13 – 7.26)
Diastolic blood pressure	High vs. low risk	1.12 (0.83 – 1.50)	1.07 (0.84 – 1.36)
	Very good	1	1
	Good	2.20 (1.11 – 4.37)	1.25 (0.76 – 2.06)
	Fair	2.38 (1.18 – 4.81)	2.18 (1.31 – 3.64)
	Poor/ very poor	6.15 (2.77 – 13.65)	3.86 (2.08 – 7.15)
Pulse rate	High vs. low risk	1.43 (1.06 – 1.93)	1.09 (0.84 – 1.42)
	Very good	1	1
	Good	2.23 (1.12 – 4.42)	1.25 (0.76 – 2.06)
	Fair	2.43 (1.2 – 4.91)	2.20 (1.32 – 3.65)
	Poor/ very poor	6.32 (2.85 – 14.03)	3.86 (2.09 – 7.14)
Low-density lipoprotein cholesterol	High vs. low risk	1.01 (0.76 – 1.35)	1.12 (0.88 – 1.44)
	Very good	1	1
	Good	2.20 (1.11 – 4.36)	1.25 (0.76 – 2.07)
	Fair	2.31 (1.14 – 4.66)	2.23 (1.34 – 3.72)
	Poor/ very poor	5.64 (2.54 – 12.56)	4.00 (2.16 – 7.39)
High-density lipoprotein cholesterol	High vs. low risk	1.70 (1.25 – 2.32)	1.08 (0.82 – 1.42)
	Very good	1	1
	Good	2.21 (1.11 – 4.38)	1.25 (0.76 – 2.06)

	**Fair**	2.38 (1.18 – 4.82)	2.20 (1.32 – 3.67)
	**Poor/ very poor**	6.17 (2.78 – 13.70)	4.01 (2.17 – 7.43)
**Triglycerides**	**High vs. low risk**	1.13 (0.81 – 1.56)	0.88 (0.65 – 1.17)
	**Very good**	1	1
	**Good**	2.20 (1.11 – 4.37)	1.28 (0.77 – 2.10)
	**Fair**	2.35 (1.16 – 4.76)	2.20 (1.32 – 3.66)
	**Poor/ very poor**	5.94 (2.66 – 13.25)	3.79 (2.05 – 7.01)
**C-reactive protein**	**High vs. low risk**	1.28 (0.92 – 1.76)	1.29 (1.00 – 1.67)
	**Very good**	1	1
	**Good**	2.23 (1.12 – 4.42)	1.27 (0.77 – 2.1)
	**Fair**	2.43 (1.20 – 4.91)	2.29 (1.38 – 3.82)
	**Poor/ very poor**	6.27 (2.83 – 13.92)	4.44 (2.4 – 8.22)
**Albumin**	**High vs. low risk**	1.16 (0.86 – 1.56)	1.83 (1.44 – 2.33)
	**Very good**	1	1
	**Good**	2.21 (1.11 – 4.38)	1.23 (0.75 – 2.03)
	**Fair**	2.38 (1.18 – 4.82)	2.18 (1.31 – 3.64)
	**Poor/ very poor**	6.11 (2.75 – 13.58)	3.82 (2.06 – 7.08)
**HbA1c**	**High vs. low risk**	1.41 (1.04 – 1.92)	1.23 (0.94 – 1.6)
	**Very good**	1	1
	**Good**	2.21 (1.12 – 4.39)	1.25 (0.76 – 2.07)
	**Fair**	2.35 (1.16 – 4.75)	2.21 (1.33 – 3.68)
	**Poor/ very poor**	5.97 (2.68 – 13.28)	3.94 (2.13 – 7.28)
**Waist-to-hip ratio**	**High vs. low risk**	1.59 (1.15 – 2.20)	1.13 (0.85 – 1.51)
