# Supplementary material for: Self-Reported Health as Predictor of Allostatic Load and All-Cause Mortality: Findings From the Lolland-Falster Health Study
Source: Int J Public Health. 2024 Feb 1;69:1606585. doi: 10.3389/ijph.2024.1606585 (PMC10866731; doi:10.3389/ijph.2024.1606585)
Supplement: Supplementary file 8 [file Table3.pdf]

Supplementary Table 3. Baseline characteristics of study population according to sex

Variable	Women					Men				
	Total	Very good	Good	Fair	Poor/ very poor	Total	Very good	Good	Fair	Poor/ very poor
**Total**	7630 (54.1)	900 (11.8)	4322 (56.6)	2061 (27.0)	347 (4.5)	6474 (45.9)	849 (13.1)	3755 (58.0)	1627 (25.1)	243 (3.8)
**Age**										
18-29	557 (7.3)	92 (16.5)	341 (61.2)	111 (19.9)	13 (2.3)	466 (7.2)	128 (27.5)	269 (57.7)	58 (12.4)	11(2.4)
30-39	649 (8.5)	69 (10.6)	389 (59.9)	161 (24.8)	30 (4.6)	514 (7.9)	60 (11.7)	325 (63.2)	120 (23.3)	9 (1.8)
40-49	1216 (15.9)	157 (12.9)	686 (56.4)	310 (25.5)	63 (5.2)	882 (13.6)	121 (13.7)	548 (62.1)	185 (21.0)	28 (3.2)
50-59	1759 (23.1)	192 (10.9)	958 (54.5)	502 (28.5)	107 (6.1)	1377 (21.3)	150 (10.9)	770 (55.9)	382 (27.7)	75 (5.4)
60-69	1908 (25.0)	210 (11.0)	1097 (57.5)	522 (27.4)	79 (4.1)	1685 (26.0)	201 (11.9)	951 (56.4)	463 (27.5)	70 (4.2)
70-79	1261 (16.5)	136 (10.8)	701 (55.6)	379 (30.1)	45 (3.6)	1252 (19.3)	142 (11.3)	727 (58.1)	341 (27.2)	42 (3.4)
80+	280 (3.7)	44 (15.7)	150 (53.6)	76 (27.1)	10 (3.6)	298 (4.6)	47 (15.8)	165 (55.4)	78 (26.2)	8 (2.7)
**Allostatic load**										
Low (0–2)	2403 (31.5)	403 (16.8)	1476 (61.4)	464 (19.3)	60 (2.5)	2119 (32.7)	356 (16.8)	1325 (62.5)	403 (19.0)	35 (1.7)
Medium (3–4)	3017 (39.5)	349 (11.6)	1754 (58.1)	796 (26.4)	118 (3.9)	2601 (40.2)	349 (13.4)	1559 (59.9)	601 (23.1)	92 (3.5)
High (5–10)	2210 (29.0)	148 (6.7)	1092 (49.4)	801 (36.2)	169 (7.6)	1754 (27.1)	144 (8.2)	871 (49.7)	623 (35.5)	116 (6.6)
**Education**										
Low	1862 (24.4)	181 (9.7)	938 (50.4)	617 (33.1)	126 (6.8)	1541 (23.8)	193 (12.5)	786 (51.0)	479 (31.1)	83 (5.4)
Medium	3475 (45.5)	373 (10.7)	1999 (57.5)	944 (27.2)	159 (4.6)	3549 (54.8)	439 (12.4)	2129 (60.0)	861 (24.3)	120 (3.4)
High	2293 (30.1)	346 (15.1)	1385 (60.4)	500 (21.8)	62 (2.7)	1384 (21.4)	217 (15.7)	840 (60.7)	287 (20.7)	40 (2.9)
**Smoking status**										
Never	3763 (49.3)	538 (14.3)	2254 (59.9)	864 (23.0)	107 (2.8)	2740 (42.3)	482 (17.6)	1667 (60.8)	527 (19.2)	64 (2.3)
Former	2464 (32.3)	264 (10.7)	1352 (54.9)	715 (29.0)	133 (5.4)	2432 (37.6)	270 (11.1)	1389 (57.1)	684 (28.1)	89 (3.7)
Current	1403 (18.4)	98 (7.0)	716 (51.0)	482 (34.4)	107 (7.6)	1302 (20.1)	97 (7.5)	699 (53.7)	416 (32.0)	90 (6.9)
**Body mass index**										
Under weight (<18.5)	140 (1.8)	23 (16.4)	67 (47.9)	42 (30.0)	8 (5.7)	42 (0.7)	6 (14.3)	19 (45.2)	12 (28.6)	5 (11.9)
Normal weight (18.5–24.9)	3172 (41.6)	539 (17.0)	1920 (60.5)	634 (20.0)	79 (2.5)	1869 (28.9)	352 (18.8)	1126 (60.2)	339 (18.1)	52 (2.8)
Over Weight (25.0–29.9)	2456 (32.2)	245 (10.0)	1445 (58.8)	656 (26.7)	110 (4.5)	2948 (45.5)	391 (13.3)	1790 (60.7)	681 (23.1)	86 (2.9)
Obese (>30.0)	1862 (24.4)	93 (5.0)	890 (47.8)	729 (39.2)	150 (8.1)	1615 (24.9)	100 (6.2)	820 (50.8)	595 (36.8)	100 (6.2)
**Cardiovascular disease**										
Yes	1954 (25.6)	129 (6.6)	988 (50.6)	703 (36.0)	134 (6.9)	2007 (31.0	129 (6.4)	1031 (51.4)	721 (35.9)	126 (6.3)
No	5676 (74.4)	771 (13.6)	3334 (58.7)	1358 (23.9)	213 (3.8)	4467 (67.0)	720 (16.1)	2724 (61.0)	906 (20.3)	117 (2.6)
**Diabetes**										
Yes	282 (3.7)	8 (2.8)	114 (40.4)	130 (46.1)	30 (10.6)	441 (6.8)	22 (5.0)	178 (40.4)	204 (46.3)	37 (8.4)
