# Supplementary material for: Self-Reported Health as Predictor of Allostatic Load and All-Cause Mortality: Findings From the Lolland-Falster Health Study
Source: Int J Public Health. 2024 Feb 1;69:1606585. doi: 10.3389/ijph.2024.1606585 (PMC10866731; doi:10.3389/ijph.2024.1606585)
Supplement: Supplementary file 9 [file Table12.pdf]

Supplementary Table 12. Difference by level of self-reported health in risk of increased level of allostatic load, ratio of relative risks (RRR) for participants including imputed data

Imputation method: Multiple imputation by chained equations.

N participants = 16016 (40 removed because of missing baseline date)

		RRR1 (95% CI)	RRR2 (95% CI)	RRR1 (95% CI)	RRR2 (95% CI)
		**Women**		**Men**	
**AL**		**Mid vs. low**	**Mid vs. low**	**Mid vs. low**	**Mid vs. low**
**SRH**	**Very good**	1	1	1	1
	**Good**	1.36 (1.16 – 1.59)	1.22 (1.04 – 1.43)	1.14 (0.97 – 1.34)	1.00 (0.85 – 1.18)
	**Fair**	1.98 (1.66 – 2.37)	1.53 (1.27 – 1.83)	1.45 (1.20 – 1.74)	1.07 (0.88 – 1.30)
	**Poor/ very poor**	2.19 (1.60 – 3.00)	1.52 (1.10 – 2.10)	2.35 (1.59 – 3.48)	1.62 (1.08 – 2.42)
**AL**		**High vs. low**	**High vs. low**	**High vs. low**	**High vs. low**
**SRH**	**Very good**	1	1	1	1
	**Good**	1.97 (1.61 – 2.42)	1.32 (1.06 – 1.65)	1.60 (1.30 – 1.98)	1.08 (0.87 – 1.35)
	**Fair**	4.82 (3.86 – 6.01)	2.09 (1.65 – 2.66)	3.70 (2.95 – 4.63)	1.59 (1.25 – 2.03)
	**Poor/ very poor**	7.50 (5.42 – 10.37)	2.38 (1.66 – 3.40)	8.57 (5.79 – 12.7)	3.10 (2.02 – 4.77)

AL = allostatic load, SRH = self-reported health
RRR1: adjusted for age at baseline.
RRR2: further adjusted for education, body mass index, smoking status, cardiovascular disease, diabetes, and cancer.
