# Supplementary material for: Self-Reported Health as Predictor of Allostatic Load and All-Cause Mortality: Findings From the Lolland-Falster Health Study
Source: Int J Public Health. 2024 Feb 1;69:1606585. doi: 10.3389/ijph.2024.1606585 (PMC10866731; doi:10.3389/ijph.2024.1606585)
Supplement: Supplementary file 10 [file Table7.pdf]

Supplementary Table 7. Multivariate Cox proportional hazard regression of all-cause mortality for participants, ratio of relative risks (RRR), adjusted for intermediate variables

	Values	Women			Men		
		**RRR 1 (95% CI)**	**RRR2 (95% CI)**	**RRR3 (95% CI)**	**RRR1 (95% CI)**	**RRR2 (95% CI)**	**RRR3 (95% CI)**
**AL**	**Medium vs. low**						
**SRH**	**Very good**	1	1	1	1	1	1
	**Good**	1.37 (1.17 – 1.61)	1.28 (1.09 – 1.51)	1.25 (1.07 – 1.47)	1.19 (1.01 – 1.40)	1.11 (0.94 – 1.31)	1.07 (0.90 – 1.26)
	**Fair**	1.98 (1.65 – 2.38)	1.72 (1.43 – 2.07)	1.63 (1.35 – 1.96)	1.50 (1.23 – 1.82)	1.30 (1.07 – 1.58)	1.21 (0.99 – 1.48)
	**Poor/ very poor**	2.27 (1.16 – 3.20)	1.87 (1.33 – 2.65)	1.74 (1.23 – 2.46)	2.65 (1.74 – 4.01)	2.31 (1.52 – 3.51)	2.10 (1.38 – 3.20)
**AL**	**High vs low**						
**SRH**	**Very good**	1	1	1	1	1	1
	**Good**	2.01 (1.64 – 2.47)	1.51 (1.22 – 1.87)	1.41 (1.14 – 1.76)	1.57 (1.27 – 1.95)	1.24 (0.99 – 1.54)	1.15 (0.92 – 1.43)
	**Fair**	4.67 (3.75 – 5.83)	2.66 (2.11 – 3.36)	2.33 (1.84 – 2.95)	3.59 (2.85 – 4.53)	2.26 (1.77 – 2.88)	1.94 (1.52 – 2.48)
	**Poor/ very poor**	7.66 (5.40 – 10.86)	3.62 (2.49 – 5.27)	2.95 (2.02 – 4.31)	7.82 (5.11 – 11.97)	4.93 (3.15 – 7.70)	3.87 (2.46 – 6.09)

AL = allostatic load
SRH = self-reported health

RRR1: Adjusted for age.
RRR2: Adjusted for age and body mass index.
RRR3: Adjusted for age, body mass index, and smoking status.
