# Supplementary material for: Self-Reported Health as Predictor of Allostatic Load and All-Cause Mortality: Findings From the Lolland-Falster Health Study
Source: Int J Public Health. 2024 Feb 1;69:1606585. doi: 10.3389/ijph.2024.1606585 (PMC10866731; doi:10.3389/ijph.2024.1606585)
Supplement: Supplementary file 11 [file Table8.pdf]

Supplementary Table 8. Multivariate Cox proportional hazard regression of all-cause mortality by using clinical cut-off values to define allostatic load

		**Women n (%)**	**HR1 (95% CI)**	**HR2 (95% CI)**	**Men n (%)**	**HR1 (95% CI)**	**HR2 (95% CI)**
			**Women**			**Men**	
**Allostatic load**	**Low (0–2)**	7442 (53%)	1	1	2788 (43%)	1	1
	**Medium (3)**	3045 (22%)	1.20 (0.84 – 1.71)	1.33 (0.93 – 1.92)	1587 (25%)	0.92 (0.67 – 1.27)	0.96 (0.69 – 1.32)
	**High (4–9)**	3617 (26%)	1.35 (0.96 – 1.89)	1.53 (1.07 – 2.18)	2099 (32%)	1.25 (0.94 – 1.65)	1.22 (0.92 – 1.63)

HR 1: Adjusted for age at baseline.
HR 2: Further adjusted for education, body mass index, smoking status, cardiovascular disease, diabetes, and cancer.
