# Supplementary material for: Self-Reported Health as Predictor of Allostatic Load and All-Cause Mortality: Findings From the Lolland-Falster Health Study
Source: Int J Public Health. 2024 Feb 1;69:1606585. doi: 10.3389/ijph.2024.1606585 (PMC10866731; doi:10.3389/ijph.2024.1606585)

Supplementary Figure 1. Kaplan–Meier curve of the survival probability according to category of the allostatic load

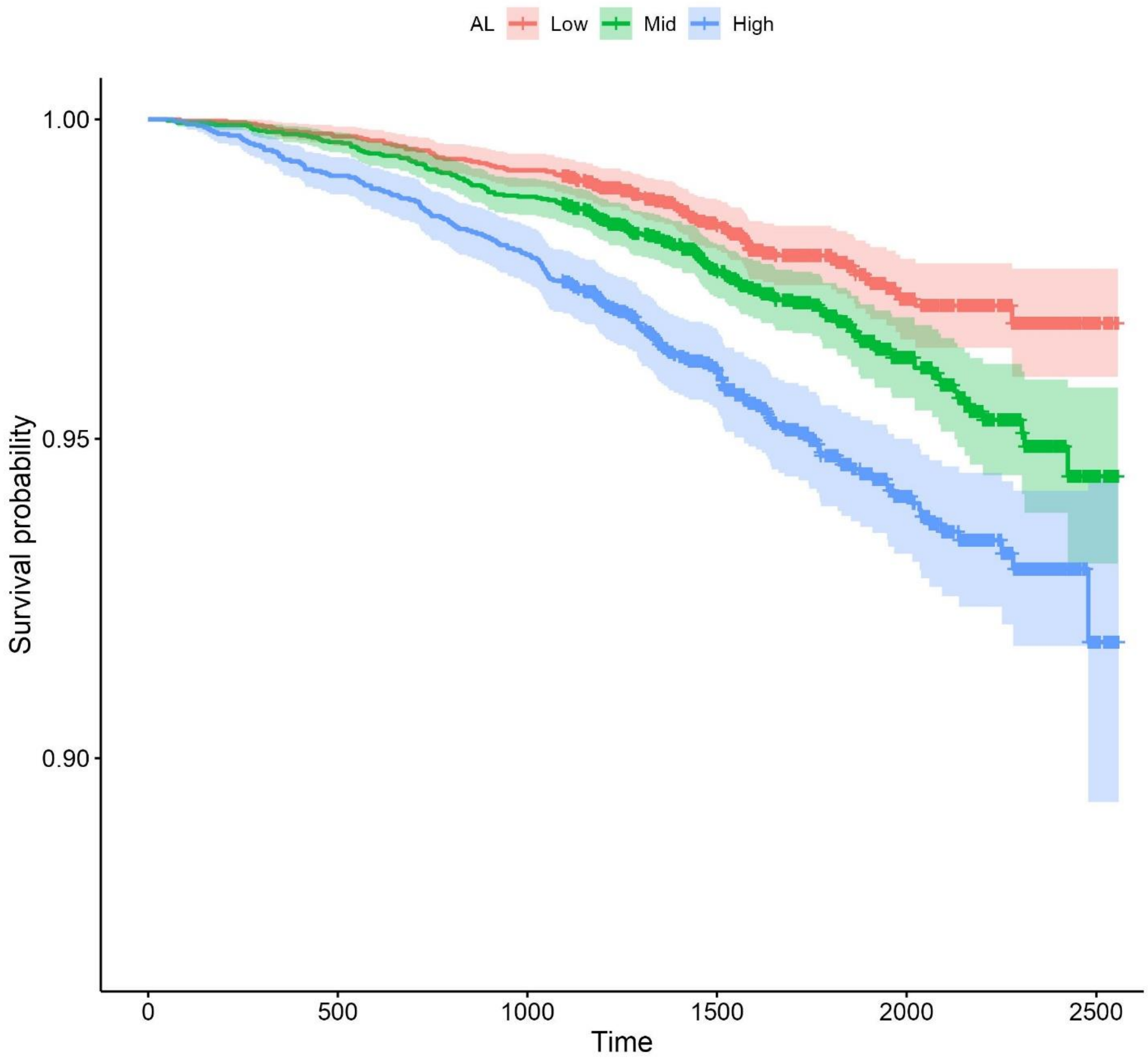

Supplement: Supplementary file 12 [file Image1.pdf]
