# Supplementary material for: Self-Reported Health as Predictor of Allostatic Load and All-Cause Mortality: Findings From the Lolland-Falster Health Study
Source: Int J Public Health. 2024 Feb 1;69:1606585. doi: 10.3389/ijph.2024.1606585 (PMC10866731; doi:10.3389/ijph.2024.1606585)
Supplement: Supplementary file 13 [file Table10.pdf]

Supplementary Table 10. Distribution of biomarkers according to self-reported health (n and %)

Variable	Characteristics	Women					Men					Total				
		Total	Very good	Good	Fair	Poor/ very poor	Total	Very good	Good	Fair	Poor/ Very poor	Total	Very good	Good	Fair	Poor/ very poor
**Total**		7630 (100)	900 (11.8)	4322 (56.6)	2061 (27.0)	347 (4.5)	6474 (100)	849 (13.1)	3755 (58.0)	1627 (25.1)	243 (3.8)	14104 (100)	1749 (12.4)	8077 (57.3)	3688 (26.1)	590 (4.2)
**SBP**																
	High risk	3932 (51.5)	449 (11.4)	2222 (56.5)	1077 (27.4)	184 (4.7)	3305 (51.1)	466 (14.1)	1907 (57.7)	800 (24.2)	132 (4.0)	7237 (51.3)	915 (12.6)	4129 (57.1)	1877 (25.9)	316 (4.4)
	Low risk	3698 (48.5)	451 (12.2)	2100 (56.8)	984 (26.6)	163 (4.4)	3169 (48.9)	383 (12.1)	1848 (58.3)	827 (26.1)	111 (3.5)	6867 (48.7)	834 (12.1)	3948 (57.5)	1811 (26.4)	274 (4.0)
**DBP**																
	High risk	4035 (52.9)	492 (12.2)	2249 (55.7)	1127 (27.9)	167 (4.1)	3304 (51.0)	460 (13.9)	1915 (58.0)	804 (24.3)	125 (3.8)	7339 (52.0)	952 (13.0)	4164 (56.7)	1931 (26.3)	292 (4.0)
	Low risk	3595 (47.1)	408 (11.3)	2073 (57.7)	934 (26.0)	180 (5.0)	3170 (49.0)	389 (12.3)	1840 (58.0)	823 (26.0)	118 (3.7)	6765 (48.0)	797 (11.8)	3913 (57.8)	1757 (26.0)	298 (4.4)
**Pulse rate**																
	High risk	2005 (26.3)	167 (8.3)	1078 (53.8)	647 (32.3)	113 (5.6)	1736 (26.8)	150 (8.6)	942 (54.3)	540 (31.1)	104 (6.0)	3741 (26.5)	317 (8.5)	2020 (54.0)	1187 (31.7)	217 (5.8)
	Low risk	5625 (73.7)	733 (13.0)	3244 (57.7)	1414 (25.1)	234 (4.2)	4738 (73.2)	699 (14.8)	2813 (59.4)	1087 (22.9)	139 (2.9)	10363 (73.5)	1432 (13.8)	6057 (58.4)	2501 (24.1)	373 (3.6)
**HDL-c**																
	High risk	2455 (32.2)	196 (8.0)	1266 (51.6)	823 (33.5)	170 (6.9)	1754 (27.1)	161 (9.2)	957 (54.6)	540 (30.8)	96 (5.5)	4209 (29.8)	357 (8.5)	2223 (52.8)	1363 (32.4)	266 (6.3)
	Low risk	5175 (67.8)	704 (13.6)	3056 (59.1)	1238 (23.9)	177 (3.4)	4720 (72.9)	688 (14.6)	2798 (59.3)	1087 (23.0)	147 (3.1)	9895 (70.2)	1392 (14.1)	5854 (59.2)	2325 (23.5)	324 (3.3)
**TG**																
	High risk	2080 (27.3)	152 (7.3)	1087 (52.3)	702 (33.8)	139 (6.7)	1698 (26.2)	172 (10.1)	922 (54.3)	512 (30.2)	92 (5.4)	3778 (26.8)	324 (8.6)	2009 (53.2)	1214 (32.1)	231 (6.1)
	Low risk	5550 (72.7)	748 (13.5)	3235 (58.3)	1359 (24.5)	208 (3.7)	4776 (73.8)	677 (14.2)	2833 (59.3)	1115 (23.3)	151 (3.2)	10326 (73.2)	1425 (13.8)	6068 (58.8)	2474 (24.0)	359 (3.5)
**WHR**																
	High risk	1915 (25.1)	138 (7.2)	951 (49.7)	674 (35.2)	152 (7.9)	1628 (25.1)	107 (6.6)	801 (49.2)	615 (37.8)	105 (6.4)	3543 (25.1)	245 (6.9)	1752 (49.4)	1289 (36.4)	257 (7.3)
	Low risk	5715 (74.9)	762 (13.3)	3371 (59.0)	1387 (24.3)	195 (3.4)	4846 (74.9)	742 (15.3)	2954 (61.0)	1012 (20.9)	138 (2.8)	10561 (74.9)	1504 (14.2)	6325 (59.9)	2399 (22.7)	333 (3.2)

**LDL-c**																
	High risk	4007 (52.5)	446 (11.1)	2280 (56.9)	1091 (27.2)	190 (4.7)	3478 (53.7)	436 (12.5)	1991 (57.2)	897 (25.8)	154 (4.4)	7485 (53.1)	882 (11.8)	4271 (57.1)	1988 (26.6)	344 (4.6)
	Low risk	3623 (47.5)	454 (12.5)	2042 (56.4)	970 (26.8)	157 (4.3)	2996 (46.3)	413 (13.8)	1764 (58.9)	730 (24.4)	89 (3.0)	6619 (46.9)	867 (13.1)	3806 (57.5)	1700 (25.7)	246 (3.7)
**HbA1c**																
	High risk	2237 (29.3)	201 (9.0)	1152 (51.5)	743 (33.2)	141 (6.3)	2024 (31.3)	178 (8.8)	1065 (52.6)	662 (32.7)	119 (5.9)	4261 (30.2)	379 (8.9)	2217 (52.0)	1405 (33.0)	260 (6.1)
	Low risk	5393 (70.7)	699 (13.0)	3170 (58.8)	1318 (24.4)	206 (3.8)	4450 (68.7)	671 (15.1)	2690 (60.4)	965 (21.7)	124 (2.8)	9843 (69.8)	1370 (13.9)	5860 (59.5)	2283 (23.2)	330 (3.4)
**CRP**																
	High risk	1909 (25.0)	143 (7.5)	943 (49.4)	674 (35.3)	149 (7.8)	1622 (25.1)	177 (10.9)	833 (51.4)	509 (31.4)	103 (6.4)	3531 (25.0)	320 (9.1)	1776 (50.3)	1183 (33.5)	252 (7.1)
	Low risk	5721 (75.0)	757 (13.2)	3379 (59.1)	1387 (24.2)	198 (3.5)	4852 (74.9)	672 (13.8)	2922 (60.2)	1118 (23.0)	140 (2.9)	10573 (75.0)	1429 (13.5)	6301 (59.6)	2505 (23.7)	338 (3.2)
**Albumin**																
	High risk	2436 (31.9)	243 (10.0)	1307 (53.7)	738 (30.3)	148 (6.1)	1804 (27.9)	217 (12.0)	1003 (55.6)	511 (28.3)	73 (4.0)	4240 (30.1)	460 (10.8)	2310 (54.5)	1249 (29.5)	221 (5.2)
	Low risk	5194 (68.1)	657 (12.6)	3015 (58.0)	1323 (25.5)	199 (3.8)	4670 (72.1)	632 (13.5)	2752 (58.9)	1116 (23.9)	170 (3.6)	9864 (69.9)	1289 (13.1)	5767 (58.5)	2439 (24.7)	369 (3.7)

SBP = Systolic blood pressure
DBP = Diastolic blood pressure
HDL-c = High-density lipoprotein cholesterol
TG = Triglycerides
WHR = Waist-to-hip ratio
LDL-c = Low-density lipoprotein cholesterol
CRP = C-reactive protein
